# Editorial: The Role of Plant Hormones in Plant-Microbe Symbioses

**DOI:** 10.3389/fpls.2019.01391

**Published:** 2019-10-30

**Authors:** Eloise Foo, Jonathan M. Plett, Juan Antonio Lopez-Raez, Dugald Reid

**Affiliations:** ^1^School of Natural Sciences, University of Tasmania, Hobart, Tasmania, Australia; ^2^Hawkesbury Institute for the Environment, Western Sydney University, Penrith, NSW, Australia; ^3^Department of Soil Microbiology and Symbiotic Systems, Experimental Station of Zaidín (EEZ), Granada, Spain; ^4^Department of Molecular Biology and Genetics, Aarhus University, Aarhus, Denmark

**Keywords:** Mycorrhizae and Rhizobium, Plant hormone, endosymbiont, ectomyccorhizas, plant associated bacteria

Plant hormones are regulators of almost all aspects of plant development and plant responses to their environment. Active at very low concentrations, with tight spatial regulation of synthesis and response, many plant hormones have key roles in the interactions between plants and beneficial microbes. In this special issue, “The Role of Plant Hormones in Plant-Microbe Symbioses,” new insights are revealed into how hormones derived from both the plant and microbial partner play roles in communication, symbioses establishment, and function. This includes intimate endosymbioses with arbuscular mycorrhizal (AM) fungi formed by the majority of land plants and the more recently evolved nodulation, the symbioses between a limited set of plants in the fabid clade and nitrogen-fixing bacteria. Articles in this special issue also explore the role of hormones in plant interactions with ectomycorrhizae, endophytic bacteria and fungi, as well as beneficial microbes that associate with the root or leaf surfaces. In addition to acting directly, many hormones can interact with each other to control the development of these symbioses, and these complex networks are now emerging.

Two review articles explore the role of hormones in AM symbioses and nodulation. Bedini et al. comprehensively examine the role of nine plant hormone groups (abscisic acid, auxins, brassinosteroids, cytokinins, ethylene, gibberellins, jasmonates, salicylic acid, and strigolactones) in AM symbiosis, including the impact of plant-derived hormones on symbiotic establishment, and their influence on plant metabolism, defense, and phosphorous status. Models are proposed that integrate hormonal signaling with plant and fungal metabolism, and future directions in specific plant priming to promote mycorrhizal performance are discussed. McGuiness et al. take a focused look at the role of gibberellins and brassinosteroids, lately emerging as important regulators of endosymbioses with AM and rhizobial bacteria. These hormones appear to play a complex role in nodulation, with both positive and negative roles proposed, which may be due to different effects of the hormones during infection and nodule organogenesis. A different story emerges for AM symbioses; gibberellins suppress AM symbiosis, while brassinosteroids appear to promote AM formation across species. Future studies examining the spatial and temporal regulation of these hormones during symbioses, including interaction with other hormones are now required.

As outlined above, symbiotic establishment and maintenance require tight regulation. Therefore, sophisticated imaging tools are required to determine the precise spatial and temporal regulation of plant hormone signaling. Nadzieja et al. describe the availability of a “toolbox” of plant imaging tools, when applied with clearing techniques to aid imaging depth, allows for analysis of hormone responses in legume roots.

The essential role for cytokinin in legume nodulation was highlighted by Gauthier-Coles et al. In a striking result, the authors found that application of cytokinin induced pseudonodules in legumes, but not non-legumes that form nodules (e.g. actinorhizal species) or other species that do not nodulate. Cytokinin-induced legume pseudonodules arose from activation of cell division in the cortex and was distinct from cytokinin regulation of other aspects of root development. This legume-specific cytokinin response offers a fascinating perspective on the evolution of nodulation in the fabid clade. As outlined above, cytokinin not only regulates nodule organogenesis but also influence rhizobial infection. Dolgikh et al. use a mutant based approach to examine how DELLA transcription factors, which act as negative regulators of gibberellin signaling, may promote early events in nodulation by influencing the expression of genes encoding cytokinin synthesis and response elements.

In contrast to its central role in nodulation, the influence of cytokinin on AM symbiosis has received considerably less attention. Studies using a pea mutant with low cytokinin level, measurement of cytokinin content, and chemical modification of cytokinin level and response by Goh et al. suggest cytokinin may play a positive role in the development of AM symbiosis. Complex regulation of cytokinin synthesis during AM colonization indicates that spatial regulation of cytokinin that has been observed during nodulation (outlined in Nadzieja et al.) may also occur during AM symbiosis, and may be an important area for future studies.

In addition to roles in uptake and establishment of the symbiotic microbe within the plant tissue, hormones may also play roles in regulating symbiotic function. Using a sophisticated set of pea mutants blocked at specific stages of nodule development, Serova et al. reveal gibberellin promotes bifurcation of the nodule meristem and nodule expansion and suppressed nodule senescence. A model of gibberellin action in nodule development suggests that early in nodule development, high gibberellin levels promotes cell cycle activation, cell division, and persistence of the nodule meristem. Conversely, during nodule aging, downregulation of active gibberellin levels leads to nodule senescence.

To deepen our understanding of the transcriptional network underpinning AM formation, Ho-Plágaro et al. performed RNA seq analysis of AM colonized tomato roots and found that more than 30% of transcription factors from the *GRAS* [acronym of gibberellin-acid insensitive (*G*AI), repressor of GA1 (*R*G*A*), and scarecrow-like (*S*CL) proteins] family were upregulated by AM symbiosis. Moreover, gene expression, promoter fusion and/or RNA interference analyses of three novel AM-regulated GRAS encoding genes revealed they may act as important players in regulating AM establishment and/or maintenance. The authors speculate that two of these genes, *SlGRAS18* and *SlGRAS38*, could interact with the GA repressor DELLA during arbuscule development and turnover.

In addition to plant-derived hormones outlined above, many plant-associated microbes also produce plant hormones, and this may have an important influence on plant partners. In bacteria, the ability to produce gibberellin appears to be limited to bacteria that associate with plants. Exploring this is more depth, Nagel et al. have recently extended our understanding of this pathway, identifying the responsible operon in a third class of proteobacteria, the beta (β)-rhizobia. Interestingly, in these bacteria the operon has lost the CYP115 enzyme, meaning that only the precursor and not active gibberellin are produced by these bacteria. The authors speculate this may have been due to the fact that gibberellin negatively influences bacterial infection of legume hosts.

Auxin or auxin precursor production by root-associated microbes or modulation of plant auxin production has been suggested as an important mechanism through which microbes regulate plant growth. Meents et al. compared the influence of beneficial and pathogenic root interacting fungi on auxin response (as measured by auxin-inducible promoter system) and hormone content of Arabidopsis. Although several of the fungi produced auxin, only one beneficial fungal endophyte (*Piriformospora indica*) appeared to rapidly and strongly induce auxin response in Arabidopsis, which was correlated with promotion of lateral root development. In contrast, auxin response was attenuated in the other interactions, and in the case of the other beneficial fungal endophyte examined (*Mortierella hyalina*), this may have been due to the production of jasmonic acid. Auxin production and 1-aminocyclopropane-1-carboxylic acid (ACC) deaminase activity, which can lower ethylene levels *in planta*, was also identified in a range of endosymbiotic bacteria isolated from two tea cultivars by Yan et al. Striking variation in the make-up of the endosymbiotic bacteria isolated both from different varieties and across seasons was also observed, suggesting complex regulation of these microbial communities. The authors propose that specific beneficial endophytes with the capacity to produce auxin and/or displaying ACC deaminase activity might assist host plant growth in unfavorable areas.

Microbial-derived hormones can also assist with plant tolerance to biotic and abiotic stress. Wagner et al. found a strong association between mycorrhizal helper bacteria (MHB), able to produce auxin and beneficial effect of ectomycorrhizae on norway spruce growth, and protection from pathogens *Botrytis cinerea* and *Heterobasidion annosum*. Similarly, plant hormone-producing fungal and bacterial endosymbionts assisted soybean to deal with heavy metal stress (Bilal et al.). In addition to promoting growth, co-inoculation with fungi *Paecilomyces formosus* and bacteria *Sphingomonas* sp. lowered Al and Zn uptake, root transport, and related oxidative stress responses in this species.

Our understanding of the role of plant hormones in beneficial plant-microbe interactions has deepened considerably over the past decade. Further research into how these hormones act in distinct cell types and at different stages of development to regulate these associations and the network of plant hormone interactions is now required. Such fundamental knowledge will aid in the implementation of such beneficial microorganisms in sustainable agriculture into the future.

## Author Contributions

EF wrote the article with contributions from JP, JALR, and DR.

## Funding

EF was supported by Australian Research Council Future Fellowship and Discovery Grants. JALR was supported by grants AGL2015-64990-C2-1R from the Spanish National R&D Plan of the Ministry of Economy and Competitiveness (MINECO) and the European Regional Development Fund (ERDF), and 201640I040 from the Spanish National Research Council (CSIC). JP would like to acknowledge the Australian Research Council for research funding (DE150100408). DR was supported by Danish National Research Foundation.

## Conflict of Interest

The authors declare that the research was conducted in the absence of any commercial or financial relationships that could be construed as a potential conflict of interest.

